# 4,4′-[Thio­phene-2,5-diylbis(ethyne-2,1-di­yl)]dibenzonitrile

**DOI:** 10.1107/S1600536808008106

**Published:** 2008-03-29

**Authors:** João Figueira, Viatslav Vertlib, João Rodrigues, Kalle Nättinen, Kari Rissanen

**Affiliations:** a Centro de Química da Madeira, LQCMM/MMRG, Departamento de Química da Universidade da Madeira, 9000-390 Funchal, Portugal; b VTT, Sinitaival 6, PO Box 1300, FI-33101 Tampere, Finland; c Nanoscience Center, Department of Chemistry, University of Jyväskylä, PO Box 35, 40014 Jyväskylä, Finland

## Abstract

In the solid state, the title compound, C_22_H_10_N_2_S, forms centrosymmetric dimers by pairs of non-classical C—H⋯S hydrogen bonds linking approximately coplanar mol­ecules. The benzene ring involved in this inter­action makes a dihedral angle of only 7.21 (16)° with the thio­phene ring, while the other benzene ring is twisted somewhat out of the plane, with a dihedral angle of 39.58 (9)°. The hydrogen-bonded dimers stack on top of each other with an inter­planar spacing of 3.44 Å. C—H⋯N hydrogen bonds link together stacks that run in approximately perpendicular directions. Each mol­ecule thus inter­acts with 12 adjacent mol­ecules, five of them approaching closer than the sum of the van der Waals radii for the relevant atoms. Optimization of the inter-stack contacts contributes to the non-planarity of the mol­ecule.

## Related literature

For related literature, see: Rodríguez *et al.* (2004 ▶, 2006 ▶); Lind *et al.* (2004 ▶); Garcia *et al.* (2001 ▶); Ornelas *et al.* (2005 ▶, 2008 ▶); Tour (2003 ▶).
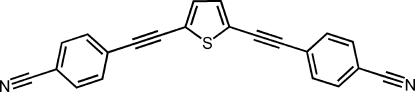


## Experimental

### 

#### Crystal data

C_22_H_10_N_2_S
*M* *_r_* = 334.38
Monoclinic, 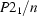

*a* = 5.4557 (11) Å
*b* = 19.467 (4) Å
*c* = 15.592 (3) Å
β = 91.89 (3)°
*V* = 1655.1 (6) Å^3^
*Z* = 4
Mo *K*α radiation
μ = 0.20 mm^−1^
*T* = 173 (2) K
0.3 × 0.2 × 0.2 mm

#### Data collection

Nonius KappaCCD diffractometer
Absorption correction: none
19547 measured reflections
2906 independent reflections
1762 reflections with *I* > 2σ(*I*)
*R*_int_ = 0.102

#### Refinement

*R*[*F* ^2^ > 2σ(*F* ^2^)] = 0.048
*wR*(*F* ^2^) = 0.107
*S* = 1.01
2906 reflections
226 parameters
H-atom parameters constrained
Δρ_max_ = 0.18 e Å^−3^
Δρ_min_ = −0.23 e Å^−3^

### 

Data collection: *COLLECT* (Hooft, 1998 ▶); cell refinement: *HKL* *SCALEPACK* (Otwinowski & Minor, 1997 ▶); data reduction: *HKL* *DENZO* (Otwinowski & Minor, 1997 ▶) and *SCALEPACK*; program(s) used to solve structure: *SHELXS97* (Sheldrick, 2008 ▶); program(s) used to refine structure: *SHELXL97* (Sheldrick, 2008 ▶); molecular graphics: *ORTEP-3 for Windows* (Farrugia, 1997 ▶) and *Mercury* (Macrae *et al.*, 2006 ▶); software used to prepare material for publication: *WinGX* (Farrugia, 1999 ▶).

## Supplementary Material

Crystal structure: contains datablocks global, I. DOI: 10.1107/S1600536808008106/cf2187sup1.cif

Structure factors: contains datablocks I. DOI: 10.1107/S1600536808008106/cf2187Isup2.hkl

Additional supplementary materials:  crystallographic information; 3D view; checkCIF report

## Figures and Tables

**Table 1:** Hydrogen-bond geometry (Å, °)

*D*—H⋯*A*	*D*—H	H⋯*A*	*D*⋯*A*	*D*—H⋯*A*
C15—H15⋯N1^i^	0.95	2.65	3.246 (4)	121
C7—H7⋯N25^ii^	0.95	2.65	3.384 (4)	134
C20—H20⋯N25^iii^	0.95	2.55	3.453 (3)	159
C5—H5⋯S12^iv^	0.95	3.05	3.832 (3)	141

Symmetry codes: (i) 
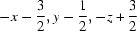
; (ii) 
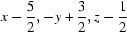
; (iii) 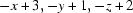; (iv) 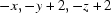.

**Fig. 1.**
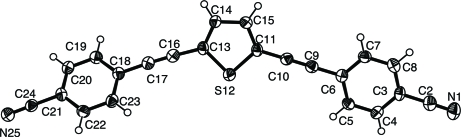
The molecular structure of (I) with 50% probability displacement ellipsoids.

**Fig. 2.**
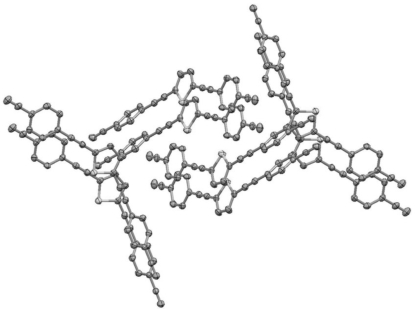
The packing of (I), viewed along the b axis.

**Fig. 3.**
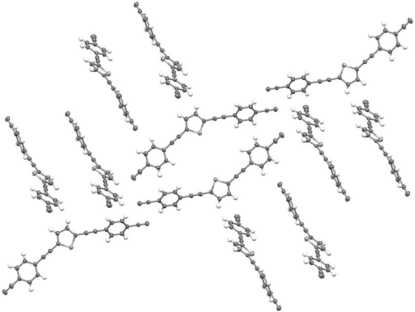
An alternate view of the packing of (I), showing the close C—H···N contacts (less that 0.1 Å + sum of vDW radii).

## supplementary crystallographic information

### Comment

The preparation of highly conjugated molecules has been of great interest for their potential applications in fields such as nanoelectronics (Tour, 2003) or optoelectronics (Ornelas *et al.*, 2005, 2008; Lind *et al.*, 2004). Terminal cyano groups provide the ability to coordinate to transition metal centres such as RuCp (Cp = cyclopentadienyl; Garcia *et al.*, 2001; Ornelas *et al.*, 2005) which should result in an increase of the physical properties such as the first molecular hyperpolarizability β, which is reported to rise with the coordination to cyclopentadienylruthenium type centres (Ornelas *et al.*, 2005, 2008). As such the preparation of the π-conjugated title compound was intended for the preparation of dinuclear ruthenium complexes for nanoelectronic application.

In the solid state the title compound, C_22_H_10_N_2_S, forms centrosymmetric dimers by pairs of non-classical C—H···S hydrogen bonds linking approximately coplanar molecules.  The benzene ring involved in this interaction makes a dihedral angle of only 7.21 (16)° with the thiophene ring, while the other benzene ring is twisted somewhat out of plane with a dihedral angle of 39.58 (9)°.  The hydrogen-bonded dimers stack on top of each other with an interplanar spacing of 3.44 Å.  C—H···N hydrogen bonds link together stacks that run in approximately perpendicular directions.  Each molecule thus interacts with twelve ajacent molecules, five of them approaching closer than the sum of van der Waals radii for the relevant atoms.  Optimisation of the inter-stack contacts contributes to the non-planarity of the molecule.

### Experimental

The title compound was prepared by Sonogashira cross-coupling (Rodríguez *et al.*, 2004, 2006) of 4-ethynylbenzonitrile (0.901 g, 7.09 mmol) and 2,5-dibromothiophene (0.800 g, 3.30 mmol) in dry tetrahydrofuran (16 ml) and *N*-ethyldiisopropylamine (25 ml). The reaction was catalysed by PdCl_2_(PPh_3_)_2_ (0.250 g, 0.360 mmol) and CuI (0.068 g, 0.36 mmol). The mixture was left under N_2_ atmosphere at room temperature for 17 h and then heated for 2. 5 h at 333–343 K. The resulting reaction mixture was washed with aqueous NH_4_Cl and extracted (3 times) with CH_2_Cl_2_. The resulting solution was dried over Na_2_SO_4_ and evaporated to dryness. The resulting dark solid was column chromatographed (Silica S60, petroleum ether/CH_2_Cl_2_ 2:2.5), yielding a pale yellow solid. Slow evaporation of a CH_2_Cl_2_ solution of the title compound resulted in yellow crystals in 41% yield. ^1^H NMR (500 MHz, CD_2_Cl_2_): δ 7.28 (2*H*, *s*, Ar); 7.62 (4*H*, *d*, Ar, J_HH_ = 9 Hz); 7.67 (4*H*, *d*, Ar, J_HH_ = 9 Hz); ^13^C NMR (126 MHz, CD_2_Cl_2_): δ 86.5, 93.4, 112.7, 118.9, 125.2, 127.8, 132.4, 132.8, 133.6, 133.7; IR (KBr): 2227 (*m*), 2207 (*m*), 1663 (*w*), 1600 (*s*), 1490 (*w*),1385 (*s*), 1110 (*w*), 865 (*s*), 839 (*s*), 802 (*m*), 555 (*m*), 536 (*w*) cm^-1^; Mp: decomposes above 393 K.

### Refinement

The H atoms were visible in electron density maps, but were placed in idealized positions and allowed to ride on their parent atoms at distances of 0.95 Å (aromatic and acetylinic), 0.98 Å (methyl) and 0.99 Å (methylene) with *U*_iso_(H) = 1.2*U*_eq_(C).

### Crystal data

**Table:**

C_22_H_10_N_2_S	*F*_000_ = 688
*M**_r_* = 334.38	*D*_x_ = 1.342 Mg m^−^^3^
Monoclinic, *P*2_1_/*n*	Mo *K*α radiation λ = 0.71073 Å
Hall symbol: -P 2yn	Cell parameters from 5465 reflections
*a* = 5.4557 (11) Å	θ = 1.0–25.0º
*b* = 19.467 (4) Å	µ = 0.20 mm^−^^1^
*c* = 15.592 (3) Å	*T* = 173 (2) K
β = 91.89 (3)º	Block, colourless
*V* = 1655.1 (6) Å^3^	0.3 × 0.2 × 0.2 mm
*Z* = 4	

### Data collection

**Table:**

Nonius KappaCCD diffractometer	*R*_int_ = 0.103
ω and φ scans	θ_max_ = 25.0º
Absorption correction: none	θ_min_ = 3.4º
19547 measured reflections	*h* = −6→6
2906 independent reflections	*k* = −23→23
1762 reflections with *I* > 2σ(*I*)	*l* = −18→18

### Refinement

**Table:**

Refinement on *F*^2^	H-atom parameters constrained
Least-squares matrix: full	*w* = 1/[σ^2^(*F*_o_^2^) + (0.0448*P*)^2^ + 0.1608*P*] where *P* = (*F*_o_^2^ + 2*F*_c_^2^)/3
*R*[*F*^2^ > 2σ(*F*^2^)] = 0.048	(Δ/σ)_max_ = 0.001
*wR*(*F*^2^) = 0.107	Δρ_max_ = 0.18 e Å^−^^3^
*S* = 1.01	Δρ_min_ = −0.23 e Å^−^^3^
2906 reflections	Extinction correction: none
226 parameters	

### Special details

**Table:**

Geometry. All e.s.d.'s (except the e.s.d. in the dihedral angle between two l.s. planes) are estimated using the full covariance matrix. The cell e.s.d.'s are taken into account individually in the estimation of e.s.d.'s in distances, angles and torsion angles; correlations between e.s.d.'s in cell parameters are only used when they are defined by crystal symmetry. An approximate (isotropic) treatment of cell e.s.d.'s is used for estimating e.s.d.'s involving l.s. planes.

### Fractional atomic coordinates and isotropic or equivalent isotropic displacement parameters (Å^2^)

**Table:**

	*x*	*y*	*z*	*U*_iso_*/*U*_eq_	
C2	−1.0858 (5)	1.19052 (14)	0.88637 (18)	0.0381 (7)	
C3	−0.9079 (4)	1.13708 (12)	0.87250 (18)	0.0321 (7)	
C4	−0.7199 (4)	1.12636 (12)	0.93351 (18)	0.0350 (7)	
H4	−0.7062	1.1548	0.9829	0.042*	
C5	−0.5532 (4)	1.07398 (13)	0.92173 (17)	0.0338 (7)	
H5	−0.426	1.066	0.9636	0.041*	
C6	−0.5711 (4)	1.03261 (12)	0.84841 (17)	0.0300 (6)	
C7	−0.7592 (4)	1.04450 (12)	0.78771 (17)	0.0341 (7)	
H7	−0.7716	1.0167	0.7376	0.041*	
C8	−0.9275 (4)	1.09612 (13)	0.79951 (18)	0.0371 (7)	
H8	−1.0561	1.1037	0.758	0.044*	
C9	−0.3976 (4)	0.97811 (13)	0.83651 (16)	0.0328 (7)	
C10	−0.2514 (4)	0.93344 (12)	0.82613 (17)	0.0318 (6)	
C11	−0.0771 (4)	0.88054 (12)	0.81401 (17)	0.0302 (6)	
C13	0.2619 (4)	0.79650 (12)	0.83234 (17)	0.0313 (6)	
C14	0.1422 (4)	0.79222 (13)	0.75409 (17)	0.0395 (7)	
H14	0.1851	0.7604	0.711	0.047*	
C15	−0.0503 (5)	0.83959 (13)	0.74389 (18)	0.0388 (7)	
H15	−0.1517	0.8428	0.6933	0.047*	
C16	0.4557 (5)	0.75490 (13)	0.86692 (17)	0.0342 (7)	
C17	0.6095 (4)	0.71891 (13)	0.89930 (17)	0.0329 (7)	
C18	0.7787 (4)	0.67373 (12)	0.94359 (17)	0.0303 (6)	
C19	0.9777 (4)	0.64472 (12)	0.90311 (17)	0.0332 (7)	
H19	1.0049	0.6553	0.8447	0.04*	
C20	1.1351 (4)	0.60097 (13)	0.94690 (17)	0.0339 (7)	
H20	1.2717	0.5819	0.919	0.041*	
C21	1.0941 (4)	0.58472 (12)	1.03170 (18)	0.0298 (6)	
C22	0.8960 (4)	0.61295 (13)	1.07333 (18)	0.0346 (7)	
H22	0.8682	0.6016	1.1315	0.041*	
C23	0.7403 (4)	0.65760 (12)	1.02936 (18)	0.0352 (7)	
H23	0.606	0.6775	1.0577	0.042*	
C24	1.2582 (5)	0.53837 (13)	1.07783 (17)	0.0330 (7)	
N1	−1.2285 (4)	1.23260 (12)	0.89832 (16)	0.0495 (7)	
N25	1.3868 (4)	0.50197 (11)	1.11512 (15)	0.0434 (6)	
S12	0.13508 (11)	0.85961 (3)	0.89414 (5)	0.0369 (2)	

### Atomic displacement parameters (Å^2^)

**Table:**

	*U*^11^	*U*^22^	*U*^33^	*U*^12^	*U*^13^	*U*^23^
C2	0.0375 (16)	0.0321 (17)	0.045 (2)	−0.0017 (13)	0.0037 (14)	0.0089 (14)
C3	0.0283 (14)	0.0245 (14)	0.0436 (19)	0.0030 (12)	0.0046 (13)	0.0035 (14)
C4	0.0353 (15)	0.0327 (16)	0.0373 (18)	0.0024 (12)	0.0045 (13)	−0.0046 (13)
C5	0.0295 (14)	0.0381 (16)	0.0332 (18)	−0.0002 (12)	−0.0059 (13)	−0.0021 (14)
C6	0.0286 (14)	0.0263 (15)	0.0352 (18)	−0.0019 (12)	0.0043 (12)	0.0030 (13)
C7	0.0371 (15)	0.0335 (16)	0.0315 (18)	0.0001 (13)	−0.0008 (13)	−0.0010 (13)
C8	0.0328 (15)	0.0383 (17)	0.040 (2)	0.0002 (13)	−0.0031 (13)	0.0054 (15)
C9	0.0314 (15)	0.0358 (16)	0.0313 (18)	−0.0013 (13)	0.0019 (12)	0.0007 (13)
C10	0.0332 (15)	0.0312 (16)	0.0310 (17)	−0.0008 (13)	0.0029 (12)	−0.0005 (13)
C11	0.0296 (14)	0.0265 (14)	0.0347 (18)	0.0017 (11)	0.0027 (12)	0.0045 (13)
C13	0.0303 (14)	0.0275 (15)	0.0363 (18)	0.0030 (12)	0.0043 (12)	0.0054 (13)
C14	0.0504 (17)	0.0369 (17)	0.0312 (19)	0.0158 (14)	0.0024 (14)	0.0009 (14)
C15	0.0492 (17)	0.0386 (17)	0.0285 (18)	0.0105 (14)	−0.0014 (13)	0.0016 (14)
C16	0.0343 (15)	0.0315 (16)	0.0369 (18)	−0.0015 (13)	0.0047 (13)	−0.0001 (14)
C17	0.0315 (15)	0.0305 (15)	0.0367 (18)	−0.0017 (13)	0.0018 (13)	−0.0007 (13)
C18	0.0304 (15)	0.0245 (14)	0.0360 (18)	−0.0026 (12)	−0.0010 (13)	−0.0010 (13)
C19	0.0339 (15)	0.0325 (16)	0.0333 (17)	−0.0018 (13)	0.0036 (13)	0.0032 (13)
C20	0.0301 (15)	0.0325 (16)	0.039 (2)	0.0028 (12)	0.0033 (13)	−0.0019 (14)
C21	0.0272 (14)	0.0254 (15)	0.0366 (19)	−0.0016 (11)	−0.0047 (12)	0.0008 (13)
C22	0.0340 (15)	0.0388 (16)	0.0308 (17)	0.0011 (13)	−0.0001 (13)	−0.0001 (13)
C23	0.0291 (14)	0.0361 (17)	0.0405 (19)	0.0032 (12)	0.0014 (13)	−0.0037 (14)
C24	0.0324 (15)	0.0314 (16)	0.0350 (18)	0.0016 (13)	−0.0026 (13)	−0.0064 (14)
N1	0.0471 (15)	0.0394 (15)	0.0626 (19)	0.0092 (12)	0.0082 (13)	0.0070 (13)
N25	0.0435 (14)	0.0459 (15)	0.0405 (16)	0.0087 (12)	−0.0057 (12)	−0.0037 (12)
S12	0.0353 (4)	0.0392 (4)	0.0360 (5)	0.0052 (3)	−0.0028 (3)	−0.0052 (3)

### Geometric parameters (Å, °)

**Table:**

C2—N1	1.149 (3)	C13—S12	1.721 (3)
C2—C3	1.444 (4)	C14—C15	1.403 (3)
C3—C8	1.391 (4)	C14—H14	0.95
C3—C4	1.392 (4)	C15—H15	0.95
C4—C5	1.383 (3)	C16—C17	1.192 (3)
C4—H4	0.95	C17—C18	1.436 (3)
C5—C6	1.399 (3)	C18—C19	1.393 (3)
C5—H5	0.95	C18—C23	1.396 (3)
C6—C7	1.392 (3)	C19—C20	1.375 (3)
C6—C9	1.438 (3)	C19—H19	0.95
C7—C8	1.378 (3)	C20—C21	1.385 (3)
C7—H7	0.95	C20—H20	0.95
C8—H8	0.95	C21—C22	1.392 (3)
C9—C10	1.195 (3)	C21—C24	1.446 (4)
C10—C11	1.418 (3)	C22—C23	1.382 (3)
C11—C15	1.365 (3)	C22—H22	0.95
C11—S12	1.724 (3)	C23—H23	0.95
C13—C14	1.367 (3)	C24—N25	1.143 (3)
C13—C16	1.424 (4)		
			
N1—C2—C3	179.1 (3)	C13—C14—H14	123.4
C8—C3—C4	120.6 (2)	C15—C14—H14	123.4
C8—C3—C2	120.1 (2)	C11—C15—C14	113.1 (2)
C4—C3—C2	119.3 (2)	C11—C15—H15	123.4
C5—C4—C3	119.5 (2)	C14—C15—H15	123.4
C5—C4—H4	120.2	C17—C16—C13	176.4 (3)
C3—C4—H4	120.2	C16—C17—C18	174.9 (3)
C4—C5—C6	120.3 (2)	C19—C18—C23	119.1 (2)
C4—C5—H5	119.8	C19—C18—C17	121.9 (2)
C6—C5—H5	119.8	C23—C18—C17	118.9 (2)
C7—C6—C5	119.3 (2)	C20—C19—C18	120.6 (2)
C7—C6—C9	120.6 (2)	C20—C19—H19	119.7
C5—C6—C9	120.1 (2)	C18—C19—H19	119.7
C8—C7—C6	120.7 (2)	C19—C20—C21	119.8 (2)
C8—C7—H7	119.7	C19—C20—H20	120.1
C6—C7—H7	119.7	C21—C20—H20	120.1
C7—C8—C3	119.6 (2)	C20—C21—C22	120.5 (2)
C7—C8—H8	120.2	C20—C21—C24	120.1 (2)
C3—C8—H8	120.2	C22—C21—C24	119.5 (2)
C10—C9—C6	179.1 (3)	C23—C22—C21	119.4 (3)
C9—C10—C11	179.8 (3)	C23—C22—H22	120.3
C15—C11—C10	128.4 (2)	C21—C22—H22	120.3
C15—C11—S12	110.82 (18)	C22—C23—C18	120.5 (2)
C10—C11—S12	120.8 (2)	C22—C23—H23	119.8
C14—C13—C16	129.1 (2)	C18—C23—H23	119.8
C14—C13—S12	110.75 (18)	N25—C24—C21	179.2 (3)
C16—C13—S12	120.1 (2)	C13—S12—C11	92.05 (12)
C13—C14—C15	113.3 (2)		
			
C8—C3—C4—C5	−0.8 (4)	C23—C18—C19—C20	−0.2 (4)
C2—C3—C4—C5	178.2 (2)	C17—C18—C19—C20	−179.2 (2)
C3—C4—C5—C6	0.9 (4)	C18—C19—C20—C21	0.9 (4)
C4—C5—C6—C7	−0.4 (4)	C19—C20—C21—C22	−0.7 (4)
C4—C5—C6—C9	−179.9 (2)	C19—C20—C21—C24	179.6 (2)
C5—C6—C7—C8	−0.4 (4)	C20—C21—C22—C23	−0.2 (4)
C9—C6—C7—C8	179.2 (2)	C24—C21—C22—C23	179.6 (2)
C6—C7—C8—C3	0.5 (4)	C21—C22—C23—C18	0.8 (4)
C4—C3—C8—C7	0.0 (4)	C19—C18—C23—C22	−0.6 (4)
C2—C3—C8—C7	−178.9 (2)	C17—C18—C23—C22	178.4 (2)
C16—C13—C14—C15	176.4 (2)	C14—C13—S12—C11	−0.5 (2)
S12—C13—C14—C15	0.1 (3)	C16—C13—S12—C11	−177.2 (2)
C10—C11—C15—C14	−179.9 (2)	C15—C11—S12—C13	0.8 (2)
S12—C11—C15—C14	−0.8 (3)	C10—C11—S12—C13	179.9 (2)
C13—C14—C15—C11	0.5 (3)		

### Hydrogen-bond geometry (Å, °)

**Table:**

*D*—H···*A*	*D*—H	H···*A*	*D*···*A*	*D*—H···*A*
C15—H15···N1^i^	0.95	2.65	3.246 (4)	121
C7—H7···N25^ii^	0.95	2.65	3.384 (4)	134
C20—H20···N25^iii^	0.95	2.55	3.453 (3)	159
C5—H5···S12^iv^	0.95	3.05	3.832 (3)	141

Symmetry codes: (i) −*x*−3/2, *y*−1/2, −*z*+3/2; (ii) *x*−5/2, −*y*+3/2, *z*−1/2; (iii) −*x*+3, −*y*+1, −*z*+2; (iv) −*x*, −*y*+2, −*z*+2.
